# Prevalence of and Risk Factors for Metabolic Syndrome, Vascular Damage, and Accelerated Aging (MetVasA) After Pediatric Hematopoietic Stem Cell Transplantation for Hematological Malignancy: Protocol for a Cross-Sectional Cohort Study

**DOI:** 10.2196/77429

**Published:** 2026-06-16

**Authors:** Lisa J Asbroek, Helena JH van der Pal, Marta Fiocco, Elizabeth AM Feijen, Lucienne H Grundeken, Maria MW Koopman, Raphaële RL van Litsenburg, Patrick van der Torre, Jan Westerink, Leontien CM Kremer, Saskia MF Pluijm, Dorine Bresters

**Affiliations:** 1 Princess Máxima Center for Pediatric Oncology Utrecht The Netherlands; 2 Mathematical Institute Leiden University Leiden The Netherlands; 3 Department of Biomedical Data Sciences Leiden University Medical Center Leiden The Netherlands; 4 Trial and Data Center Princess Máxima Center for Pediatric Oncology Utrecht The Netherlands; 5 Department of Internal Medicine Isala Hospital Zwolle The Netherlands; 6 Wilhemina Children's Hospital University Medical Center Utrecht Utrecht The Netherlands

**Keywords:** metabolic syndrome, endothelial dysfunction, vascular damage, accelerated aging, health behaviors, survivors of childhood cancer, hematopoietic (stem) cell transplantation, late effects, pediatric oncology

## Abstract

**Background:**

Survivors of hematopoietic stem cell transplantation (HSCT) in childhood face a high risk of metabolic and cardiovascular disease as well as accelerated aging. Estimates of the prevalence of these severe late effects have varied widely because they have been based on small cohorts or mixed populations of patients that received transplantations for both malignant and nonmalignant diseases, and the co-occurrence of these late effects was not assessed. Therefore, the true burden of these complications in survivors of hematological malignancies remains unclear. Moreover, the role of potentially modifiable risk factors such as health behaviors and inflammation has not yet been determined.

**Objective:**

This study aims to determine the prevalence of metabolic syndrome (MetS), endothelial dysfunction (ED), and accelerated aging and their risk factors, including treatment-related factors, inflammation, and health behaviors, in a large representative cohort of Dutch survivors of HSCT in childhood. Additionally, the study will examine the co-occurrence of these late effects.

**Methods:**

This cross-sectional cohort study will combine 2 cohorts of survivors of HSCT in childhood for a hematological malignancy. The first cohort (cohort 1; n=102) consists of survivors who received transplantations before 2002 and were participants of the Dutch Childhood Cancer Survivor Study LATER 2 cohort, a nationwide cohort study focusing on late effects among long-term childhood cancer survivors. The second cohort will include survivors who received transplantations between 2002 and 2021 (cohort 2; projected n=120) and visited the late effects (LATER) outpatient clinic of the Princess Máxima Center between 2024 and 2026. Key outcomes will be the prevalence of MetS (≥3 of 5 clinical criteria), accelerated aging (3 of 5 biological and clinical criteria), and ED (assessed by endothelial peripheral arterial tonometry) and their co-occurrence. A broad range of potential and modifiable risk factors will be investigated, including treatment-related factors; transplant complications (eg, graft-versus-host disease); and health behaviors, including physical activity, dietary intake and status assessed with nutritional biomarkers, substance use, sun exposure, and relaxation.

**Results:**

Patient recruitment started in January 2024 and is estimated to last until June 2026. As of September 2025, a total of 77 participants have been included in the study.

**Conclusions:**

This study will provide insight into the prevalence of MetS, ED, and accelerated aging, as well as potentially modifiable risk factors, including those that have not been previously examined, among survivors of HSCT for hematological malignancies in childhood. The findings will inform surveillance guidelines and support the development of health behavioral and anti-inflammatory interventions to mitigate the risk of these severe late effects.

**International Registered Report Identifier (IRRID):**

DERR1-10.2196/77429

## Introduction

Allogeneic hematopoietic stem cell transplantation (HSCT) offers a potentially curative treatment for some children with a hematological malignancy and has been increasingly used over the past decades [1]. Advances in stem cell availability, treatment modalities, and supportive care have significantly improved survival rates after HSCT [2]. However, HSCT remains an intensive treatment with a substantial burden of late effects. Survivors of HSCT face a higher risk of severe late effects as compared to patients treated with chemotherapy alone, which may negatively impact their quality of life [3]. Metabolic syndrome (MetS) and vascular disease are among the most frequently observed late effects [3-12].

Survivors of HSCT have a high risk of developing MetS or its components, including overweight, insulin resistance, and diabetes mellitus [13-24]. The estimated prevalence of MetS in survivors of HSCT ranges from 23% to 49%, whereas in the general Dutch population aged 30 to 40 years and 40 to 50 years, the prevalence is 14% and 24%, respectively [25]. MetS is strongly associated with a high risk of developing diabetes mellitus and cardiovascular disease (CVD) [26].

CVD is another frequent and potentially severe late effect in survivors of HSCT, with cumulative incidences ranging from 4% to 48% after a median follow-up of 3 to 25 years [21,27-31]. Endothelial dysfunction (ED) can be an early sign of CVD and thus predicts an increased risk for cardiovascular morbidity [32,33].

In addition to vascular and metabolic complications, survivors of HSCT often experience multiple other aging-related morbidities in young or middle-age adulthood that are typically seen in the older general population. Frailty, an aging phenotype primarily described in older adults, is over 4 times more prevalent in survivors of HSCT than in survivors treated with chemotherapy alone [34]. This indicates that a process of accelerated aging occurs, which has also been suggested by other studies in survivors of HSCT [3,6,35-38]. This phenomenon impairs quality of life and predisposes survivors of HSCT to early morbidity and mortality.

**Figure 1:**
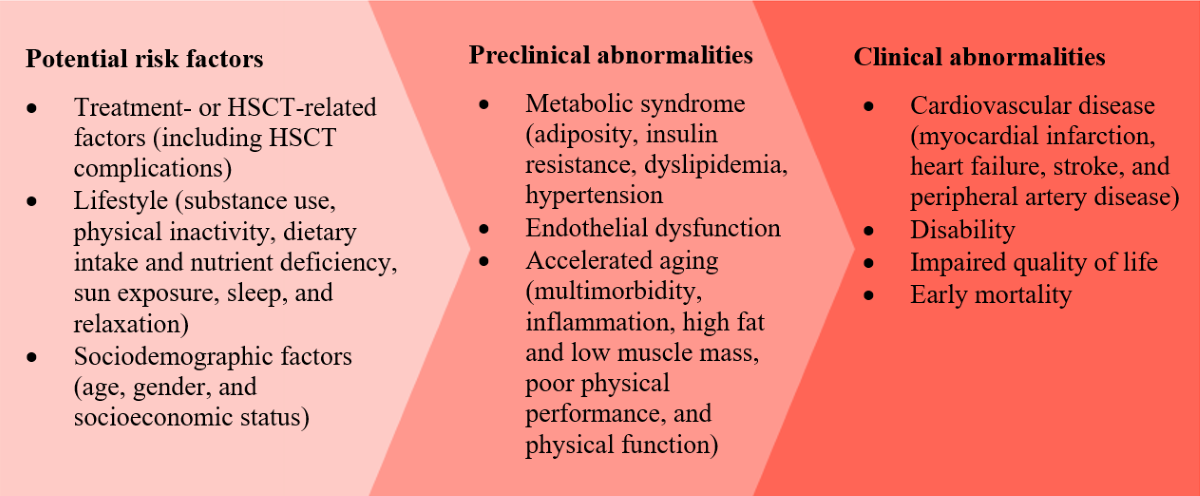
Potential risk factors for and consequences of metabolic syndrome, endothelial dysfunction, and accelerated aging. HSCT: hematopoietic stem cell transplantation.

Vascular and metabolic diseases are linked through shared pathophysiologic pathways [39]. Similarly, graft-versus-host disease (GvHD) and ED syndromes share a common pathophysiology that is characterized by tissue damage, cytokine release, and inflammation with endothelial injury [40-43]. HSCT is characterized by severe tissue damage caused by the conditioning regimen, along with severe immunosuppression in the initial phase, which is often complicated by infectious problems. Together, these factors lead to an inflammatory milieu. Engraftment and immune reconstitution may trigger immune dysregulation and inflammation, potentially leading to acute GvHD and further amplifying this inflammatory state [44-49]. Chronic GvHD and its treatment are also identified as important risk factors for the development of late effects and may contribute to long-term inflammation [7,9,10,50,51]. The conditioning regimen, immune dysregulation, inflammation, GvHD and its treatment, as well as infection or viral reactivation may contribute to endothelial damage. Moreover, posttransplant ED syndromes are characterized by endothelial activation and damage [40-42]. MetS, ED, and aging may also be linked to several predisposing unhealthy lifestyle behaviors such as sedentary behavior, low levels of physical activity, and unbalanced dietary patterns [52-56]. The co-occurrence of MetS, ED, and other aging-related hallmarks predispose survivors of HSCT to CVD, disability, impaired quality of life, and early mortality (Figure 1).

Up until now, studies investigating late effects in childhood survivors of HSCT have been limited by small sample sizes and heterogeneous populations, often including survivors who received transplantations for malignant diseases as well as those whose transplantations were for nonmalignant disease. Thus, the estimates of the prevalence of these late effects have been imprecise and not specific to survivors of hematological malignancies. There is a need to better understand these late effects and their associated risk factors.

Late effects after HSCT likely result from a combination of risk factors related to the treatment and transplant, lifestyle behavior, and sociodemographics [7,9,10,50,51,57]. Although previous studies examined the conditioning regimen and total body irradiation as risk factors for developing MetS or vascular disease in survivors of HSCT, lifestyle behaviors or HSCT-related complications (eg, chronic GvHD and ED syndromes) have not been examined so far [14,26,58]. Identifying (potentially modifiable) risk factors, including lifestyle behaviors, may aid in developing targeted prevention strategies for those with the highest risk of developing these late effects [35,59,60].

The Metabolic Syndrome, Vascular Damage, and Accelerated Aging (MetVasA) study aims to provide a comprehensive analysis of MetS, ED (as an early sign of vascular disease), and accelerated aging in a nationwide cohort of survivors of HSCT for a hematological malignancy in childhood. The primary aims of this study are to determine the prevalence of and risk factors for (1) MetS, (2) ED, and (3) accelerated aging. Transplant-related factors (eg, conditioning regimen, donor type, and stem cell source), transplant-related complications (eg, ED syndromes and GvHD), sociodemographic factors, and lifestyle behaviors will be examined. Second, we will determine the (4) coexistence of MetS, ED, and accelerated aging and their combined risk factors, and (5) prevalence of unhealthy lifestyle behaviors, including smoking, excessive alcohol consumption, other substance use, low level of physical activity, poor quality and/or quantity of sleep, and poor dietary intake of vitamins and nutrients.

## Methods

### Study Design and Population

#### Overview

**Figure 2:**
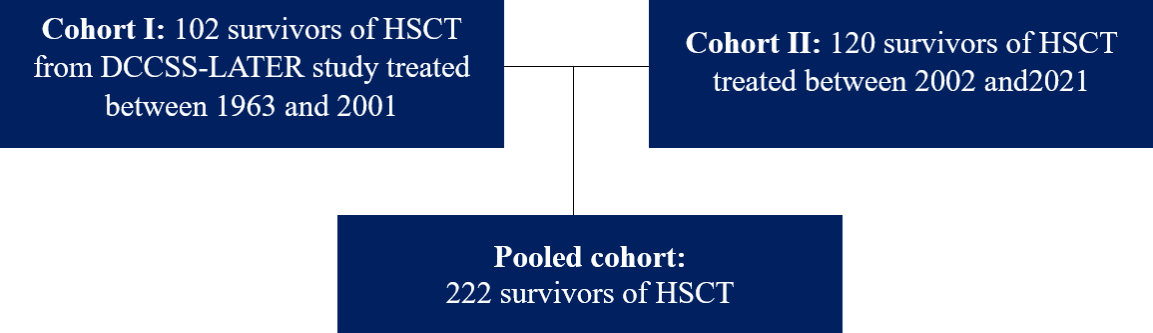
Overview of cohorts for the Metabolic Syndrome, Vascular Damage, and Accelerated Aging study. DCCSS-LATER: Dutch Childhood Cancer Survivor Study-LATER; HSCT: hematopoietic stem cell transplantation.

The MetVasA study is a cross-sectional study comprising 2 cohorts of long-term survivors of HSCT who underwent HSCT for a hematological malignancy during childhood. Data of the first cohort were previously collected as part of the Dutch Childhood Cancer Survivor Study (DCCSS) LATER 2 study (cohort 1). A second cohort, consisting of more recently treated survivors of HSCT, will be invited for this study at the late effects (LATER) outpatient clinic of the Princess Máxima Center for Pediatric Oncology in Utrecht, the Netherlands (cohort 2). To enhance statistical power, data from both cohorts will be pooled (Figure 2).

#### Cohort 1: DCCSS-LATER 2 Study Participants

The DCCSS-LATER study cohort is a national cohort of childhood survivors of cancer and includes >6000 survivors diagnosed between January 1, 1963, and December 31, 2001, before the age of 18 years, who survived at least 5 years after diagnosis and were treated in 1 of the 7 pediatric oncology centers in the Netherlands. The DCCSS-LATER study comprises 2 parts. Part 1 consists of a general health and health behavior questionnaire. Part 2 (from 2016 to 2020) includes the evaluation of a broad spectrum of late effects and possible risk factors using diagnostic methods such as blood samples, imaging studies, and organ function assessments. The methodology of this study has been described in more detail previously [61]. A representative sample of 102 survivors of HSCT participated in the DCCSS-LATER 2 study and the DCCSS-LATER 2 substudies on MetS and aging.

#### Cohort 2: Survivors of HSCT From 2002 to 2021

A second, newly established cohort will include both adult survivors as well as survivors aged <18 years, who underwent HSCT in childhood (aged ≤18 years at the time of HSCT) for a hematological malignancy between January 1, 2002, and January 1, 2021. Eligible survivors must be alive for at least 2 years after transplant and currently receiving follow-up care at the LATER outpatient clinic of the Princess Máxima Center for Pediatric Oncology. Participants must be at least 4 years old at the time of inclusion in this study to ensure they can comply with study procedures. This cohort consists of approximately 255 survivors, of which we aim to include 120 survivors based on the power calculation (refer to the Power calculation section). Exclusion criteria include active treatment for a relapse or secondary malignancy, (possible) pregnancy, and insufficient proficiency in Dutch language or illiteracy.

Every month, the coordinating researcher will receive a list of eligible participants scheduled for routine follow-up appointments at the late effects outpatient clinic of the Princess Máxima Center for Pediatric Oncology in the upcoming months. The coordinating researcher contacts the eligible participants before their regular scheduled follow-up visit to inform them about the study and invite them to participate. If they agree to participate, written informed consent will be obtained.

To assess the representativeness of the final study sample, aggregated data on the total eligible cohort will be obtained from the Dutch Childhood Cancer Survivor Registry (DCCSR).

### Data Collection

Data and samples of cohort 1 were previously collected in the DCCSR. For participants in cohort 2, a study visit will be scheduled to coincide with their regular follow-up care visit to the LATER outpatient clinic in the Princess Máxima Center to minimize participant burden. During this study visit, a venous blood sample will be collected simultaneously with the blood draw performed for their regular follow-up care. Furthermore, anthropometric measurements and several clinical tests will be performed, as discussed in Assessment of Outcomes. In addition to the study visit, participants will be asked to complete online questionnaires. For participants aged <12 years, the legal guardian will be requested to complete the questionnaires together with the participant. Furthermore, all participants will be asked to complete a home-based measurement week within 3 months after their study visit, as discussed in Assessment of Health Behaviors.

Detailed information on the type of hematological malignancy and treatment for the primary disease and any recurrences will be retrieved from the DCCSR. Data on HSCT characteristics (eg, condition regimen, donor type, and stem cell source) and post-HSCT complications (eg, ED syndromes and GvHD) will be extracted from medical records.

### Assessment of Outcomes

#### Assessment of MetS and Its Components

We will assess MetS and its components in participants of both cohorts using the modified National Cholesterol Education Program Adult Treatment Panel III criteria for participants aged ≥16 years [62]. For children aged <16 years, there is no consensus on the cutoff values for each criterion. On the basis of existing literature, we will use the criteria proposed by Ahrens et al [63] for children aged 4 to 10 years (Identification and Prevention of Dietary- and Lifestyle-Induced Health Effects in Children and Infants criteria) [64]. As the Identification and Prevention of Dietary- and Lifestyle-Induced Health Effects in Children and Infants study provided no reference values for the homeostatic model assessment of insulin resistance, triglycerides, and high-density lipoprotein cholesterol for children aged ≥11 years, , we will use the National Cholesterol Education Program Adult Treatment Panel III pediatric criteria for participants aged 11 to 16 years (Table 1) [65]. Adiposity will be assessed by waist circumference (WC; measured at the middle between the lower rib and iliac crest to the nearest centimeter) and body fat *t* score or *z* score (assessed by dual-energy x-ray absorptiometry [DXA]). Insulin resistance will be assessed by fasting glucose and/or with the Homeostatic Model Assessment of Insulin Resistance ([fasting insulin (µU/mL)×fasting glucose (mmol/L)]/22.5). Dyslipidemia will be assessed by levels of triglycerides and high-density lipoprotein cholesterol. Hypertension will be assessed by the mean of 2 blood pressure measurements in a sitting position using an electronic oscillometric meter, with 5 minutes of rest between the 2 measurements. Medical records will be checked for the current use of medication for either diabetes mellitus, hypertension, or dyslipidemia.

**Table 1:** Indicators, definitions, and criteria for determination of metabolic syndrome.

			NCEP ATP-III^a^ adult criteria (2005; aged ≥16 years)^b^		NCEP ATP III pediatric criteria (2005; aged 11-16 years)^b^		IDEFICS^c^ criteria (aged ≤10 years)^b^		Alternative with DXA^d^-scan^e^
**Adiposity**									
	Waist circumference (cm)	>102^f^/88^g^		≥90th percentile (age- and sex-specific)		≥90th percentile (age- and sex-specific)		Body fat *t* score/*z* score>2	
**Insulin resistance**									
	Fasting glucose (mmol/L)	≥5.6 or treatment		≥5.6 or treatment		≥90th percentile or HOMA-IR^h^ ≥90th percentile or treatment		—^i^	
**Dyslipidemia**									
	Triglycerides (mmol/L)	≥1.7 or treatment		≥1.2 or treatment		≥90th percentile or treatment		—	
	HDL^j^ cholesterol (mmol/L)	<1.0^f^/1.3^g^ or treatment		<1.0 or treatment		≤10th percentile or treatment		—	
**Hypertension**									
	Blood pressure (mmHg)	≥130/85 or treatment		≥90th percentile of SBP^k^ or DBP^l^ (age-, sex-, and height-specific) or treatment		≥90th percentile of SBP or DBP (age-, sex-, and height-specific) or treatment		—	

^a^NCEP ATP III: National Cholesterol Education Program Adult Treatment Panel III.

^b^≥3 of the components in each column are required for diagnosis.

^c^IDEFICS: Identification and Prevention of Dietary- and Lifestyle-Induced Health Effects in Children and Infants.

^d^DXA: dual-energy x-ray absorptiometry.

^e^Adiposity assessed by body fat *t* score/z score was used instead of waist circumference in the diagnosis of metabolic syndrome to explore potential underestimation of adiposity by waist circumference in this cohort.

^f^Men.

^g^Women.

^h^HOMA-IR: homeostatic model assessment for insulin resistance.

^i^Not applicable.

^j^HDL: high-density lipoprotein.

^k^SBP: systolic blood pressure.

^l^DBP: diastolic blood pressure.

#### Assessment of ED

Endothelial function will be assessed using a standardized procedure with endothelial peripheral arterial tonometry using the EndoPAT2000 (Itamar Medical, Caesarea, Israel) in accordance with the instruction manual [66]. This assessment will only be performed in cohort 2. ED established by endothelial peripheral arterial tonometry has been shown to be a simple and reliable measurement technique to assess endothelial function in adults and adolescents [67-69]. To define an abnormal reactive hyperemia index (RHI) in adults, the validated cutoff point of the RHI of 1.35 will be used. Age-specific normal values of the RHI have not been established for children. Median values that have been reported in other studies for children aged 12 to 18 years are 2.15 for lean children with normal lipid levels and 1.50 for severely obese children. Therefore, for children, we will use an RHI cutoff value of 1.50.

#### Assessment of Accelerated Aging and Its Indicators

##### Overview

**Figure 3:**
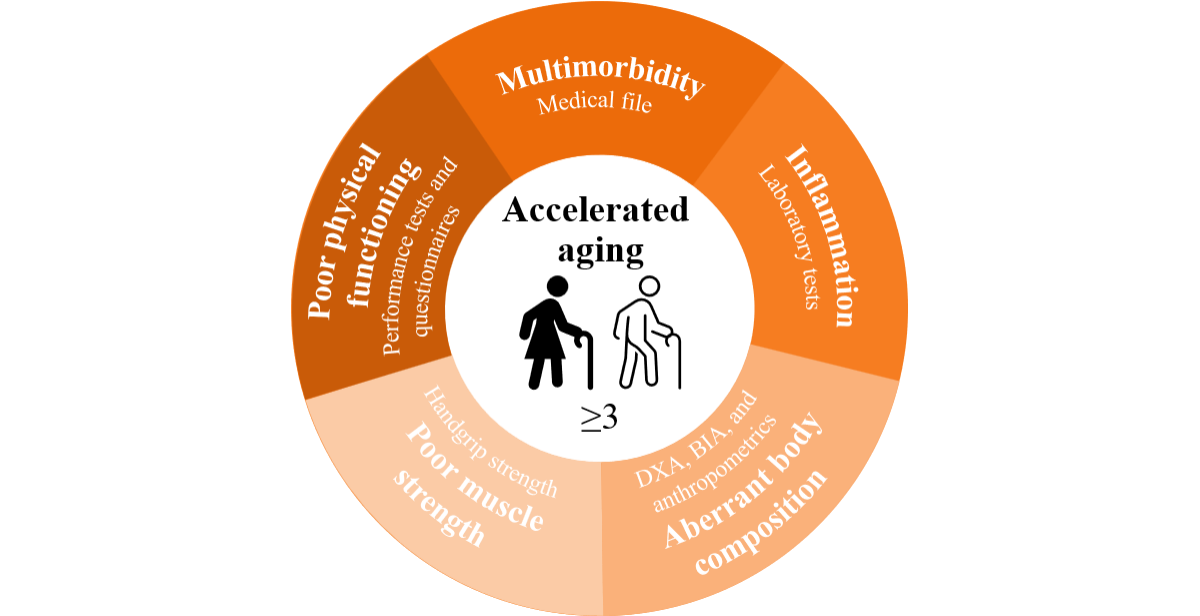
Aging indicators. BIA: bioimpedance analysis; DXA: dual-energy x-ray absorptiometry.

For assessing accelerated aging, we composed a comprehensive score with five indicators: (1) multimorbidity, (2) inflammation, (3) aberrant body composition, (4) poor muscle strength, and (5) poor physical function. We define accelerated aging as the presence of at least 3 of 5 indicators. Figure 3 shows an overview of the assessment of these indicators.

##### Multimorbidity

Multimorbidity will be assessed with a questionnaire and data retrieved from the medical records regarding the presence, type, and number of chronic diseases and medication use. The 18-item Pediatric Quality of Life Inventory 3.0 Multidimensional Fatigue Scale will be used to assess fatigue [70]. Multimorbidity will be defined as the coexistence of ≥2 age-related chronic diseases, including but not limited to fatigue, renal and heart failure, pulmonary and musculoskeletal diseases, diabetes, osteoporosis, and subsequent malignant neoplasms.

##### Inflammation

Inflammation will be assessed by analysis of inflammatory biomarkers in blood samples, including high sensitivity C-reactive protein, tumor necrosis factor-α, interleukin-6, and interleukin-10. Inflammation will be defined as the presence of ≥2 elevated inflammatory markers.

##### Aberrant Body Composition

Body composition was assessed by DXA and anthropometry in cohort 1. DXA was used to establish the amount of appendicular lean body mass, total fat, bone mass, and bone mineral density. *Z* scores will be calculated for lean body mass, fat mass, and bone mineral density. Furthermore, BMI (kg/m^2^), calculated from body weight (kg) measured with a beam scale to the nearest 0.1 kg and body height (cm) assessed with a stadiometer without shoes to the nearest centimeter, and WC, as described for MetS, were assessed. For cohort 2, body composition will be assessed similarly with DXA and anthropometric measurements. However, we will also perform a bioimpedance analysis (Tanita MC700A). Bioimpedance analysis is an inexpensive, accessible, user-friendly, noninvasive, and reliable method in which body composition is assessed by measuring bioelectrical resistance [71]. We will calculate *z* scores for lean body mass, fat mass, and bone mass. Their agreement with DXA will also be assessed. We will score body composition on low muscle mass, underweight, and adiposity. Low muscle mass will be defined as a lean body mass *z* score ≤2. Underweight will be defined as fat mass *z* score ≤2 or a BMI <18.5 for participants aged ≥19 years and BMI <17.0 for participants aged <19 years. Adiposity will be defined as fat mass *z* score >2 or increased WC using the same cutoff values as for MetS (Table 1). Additionally, the Body Roundness Index will be calculated (364.2 − 365.5 × {1−[(WC / 2π) / (0.5 × height)]}). The Body Roundness Index might be a better predictor for MetS than BMI and WC, as this is a predictor for body fat percentage and visceral adiposity tissue [72].

##### Poor Muscle Strength

Muscle strength was or will be assessed in both cohorts by handgrip strength using a handheld dynamometer (Jamar plus). Participants were or will be asked to perform 2 maximum force trials with each hand. The maximum of the right and left hand will be summed and used as the final score. The hand grip strength will be scored as poor with a score <10^th^% based on reference data.

##### Poor Physical Function

In cohort 1, physical functioning was assessed using the 6-minute-walking test (6MWT) [73,74, 75], a submaximal test of aerobic capacity where participants were encouraged to walk as fast as possible for 6 minutes along a 20-m track. The total distance covered in 6 minutes was recorded as the score. Additionally, self-reported physical function was evaluated using the physical function and vitality subscales of the Medical Outcome Study 36-item short form questionnaire [76].

In cohort 2, physical performance will be assessed more comprehensively. As in cohort 1, self-reported physical function will be assessed using the physical function and vitality subscales of the Medical Outcome Study 36-item short form questionnaire. Participants will also be asked to complete the Tilburg Frailty Index [77]. Additionally, several aspects of physical performance will be assessed with test batteries tailored to different age groups.

For participants aged <18 years, physical performance will be assessed using the Eurofit test battery, which includes various health-related and skill-related fitness tests [78]: Flamingo balance test (balance), plate tapping (upper body speed), sit-and-reach (flexibility), standing broad jump (lower body muscular strength), handgrip strength (upper body muscular strength), sit-ups (abdominal muscular endurance), bent arm hang (upper body muscular endurance), 10×5-m agility shuttle run (running speed-agility), and a 20-m shuttle run (cardiorespiratory fitness).

For participants aged ≥18 years, physical performance will be assessed using the Short Physical Performance Battery [74,79], handgrip strength [80], 6MWT, and the steep ramp test (SRT) [81-83]. The 6MWT is described above. The Short Physical Performance Battery includes a timed 4-m walk (gait speed), chair sit-to-stand test (lower body muscular strength), and standing balance tests (total score 0-12). The SRT is performed on a calibrated cycle ergometer on which the work rate (wattage) is linearly increased by a ramp protocol, and participants need to maintain a minimal number of revolutions per minute (60 rpm) up to a maximal achieved wattage. The SRT performance is strongly associated with peak oxygen uptake and provides a reliable indication of cardiorespiratory fitness.

#### Assessment of Health Behaviors

The health behaviors of the participants will be assessed with questionnaires and laboratory tests in both cohorts.

In cohort 1, participants completed the Short Questionnaire to Assess Health-Enhancing Physical Activity to assess the level of physical activity [84]. In cohort 2, the Short Questionnaire to Assess Health-Enhancing Physical Activity was also used, but additional assessments were conducted using the Patient-Reported Outcomes Measurement Information System Sleep Disturbance 8-item short form [85,86] and a more comprehensive questionnaire on health behaviors that includes substance use (smoking, alcohol consumption, and drug use), supplement use, dietary intake, meditation, mindfulness, and sun exposure.

In cohort 2, participants will undergo a measurement week using 2 accelerometers (ActiGraph wGT3x-BT) to measure sleep quantity and quality, as well as frequency and intensity of physical activity [87]. Additionally, participants will be requested to keep a diary documenting if the accelerometers were temporarily removed, which physical activity they engaged in, and whether they took a nap during the day. During this measurement week, participants will also track their nutrition for 3 days using the Eetmeter app (Voedingscentrum). Data from the Eetmeter app, combined with the questionnaire on health behaviors, will be analyzed using the Dutch Healthy Diet Index [88,89].

In both cohorts, laboratory tests to assess nutritional status and nutrient levels will be performed.

### Power Calculation

Power calculations were conducted for all outcomes. The sample size is based on the power calculation for MetS, as this is our primary outcome. Assuming the population proportion under the null hypothesis of 20% and under the alternative hypothesis of 28%, calculation performed using G*Power indicated that a sample size of n=222 is needed to detect a difference of 8% between the alternative hypothesis and the null hypothesis using a 2-sided binomial test with 80% power. The target significance is *P*=.05, and actual significance level achieved is *P*=.04. We already have a cohort (cohort 1) of 102 survivors, so we will need to include 120 additional survivors in cohort 2. With a total sample size of 222 survivors and an expected prevalence of 0.28 for MetS, the 95% CI for the estimated proportion will be 0.22-0.34.

### Statistical Analysis

SPSS Statistics (version 29.0.0; IBM Corp) will be used for statistical analyses. Categorical data will be summarized as frequencies and percentages. For continuous data, the mean, SD, and median will be presented. Descriptive statistics will be used to compare participants with and without MetS, ED, or accelerated aging. A 2-sided binomial test will be used to compare the prevalence of MetS in the different groups. The prevalence will be estimated within categories of attained age, sex, and follow-up time. Additionally, multivariable regression analysis will be used to adjust the estimated prevalence for the entire cohort, accounting for age at diagnosis, sex, attained age, and/or follow-up time. Estimated prevalences with 95% CI will be presented. Furthermore, a sensitivity analysis will be conducted, in which the prevalence with and without participants with <5 years of follow-up will be compared. Student *t* test (for normally distributed continuous variables), Mann-Whitney *U* test (for skewed continuous variables), and *χ*^2^ test (for categorical variables) will be used to compare participants and nonparticipants (those who were eligible but did not participate).

To determine the association between potential risk factors (treatment-related factors, HSCT-related factors, sociodemographics, and unhealthy lifestyle behaviors) and MetS, ED, and accelerated aging, univariable and multivariable logistic regression models will be estimated. Odds ratios with 95% CI will be reported.

Univariable and multivariable multinomial models will be estimated to study the association between independent variables and the co-occurrence of MetS, ED, and accelerated aging. The outcome will be categorized as having (1) 1 late effect (either MetS, ED, or accelerated aging), (2) 2 late effects (any combination of MetS, ED, and accelerated aging), and (3) all 3 late effects.

### Ethical Considerations

The MetVasA study was approved by the Central Medical Research Ethics Committee (NedMec) in June 2023 and is registered at the Central Committee on Research Involving Human Subjects (NL83998.041.23). In addition, the study received approval from the local Research Ethics Committee at the Princess Máxima Center for Pediatric Oncology. Participants in cohort 1 have previously provided written informed consent in the context of the DCCSS-LATER 2 study. Permission for secondary analyses without the need for additional consent was included in this initial consent and is covered under the current ethical approval. For all participants in cohort 2, written informed consent will be obtained. Participants receive detailed information about the purpose, procedures, risks, and benefits of the study prior to participation, and are informed about their right to withdraw their informed consent at any time without negative repercussions and the obligation to provide a reason; if a reason is given, it will be documented. To assess feasibility and acceptability, inclusion and dropout rate will be monitored closely. To protect participant confidentiality, all data will be deidentified prior to analysis. Participants do not receive any financial compensation for participation.

## Results

Funding for this study was obtained in October 2022, and the first participant was included in January 2024. As of September 2025, a total of 77 participants have been included in the study and completed the study visit. The recruitment of participants is expected to continue until June 2026. The results of this study are expected to be published between late 2026 and early 2027.

## Discussion

In the MetVasA study, we will assess the prevalence of and risk factors for MetS, ED, and accelerated aging in survivors of HSCT in childhood for a hematological malignancy, as well as the prevalence of an unhealthy lifestyle in these survivors. Finally, we will explore the co-occurrence of MetS, ED, and accelerated aging and its risk factors. As of September 2025, a total of 77 participants have already been included in cohort 2 and completed their clinic visit. Inclusion is estimated to continue until June 2026.

This study has several strengths, including the availability of a large representative national cohort of survivors of HSCT; the thoroughness of the collected data; and the clinical evaluation of components of MetS, ED, and accelerated aging in addition to self-reported outcomes assessed with validated questionnaires. To our knowledge, this is the first study to examine metabolic, vascular effects, and aging late effects together, as well as to analyze HSCT complications such as GvHD and ED syndromes as risk factors for these outcomes. Moreover, it is the first study to investigate potentially modifiable health behaviors in survivors of HSCT.

However, the extensiveness of data collection brings challenges in participant recruitment and achieving a complete dataset. Participating in the study requires a significant time investment from participants, and some may find the burden too great or may forget to complete certain study parts. To address this, we will regularly track the completeness of the data and discuss strategies to motivate participants to complete all required assessments.

In conclusion, this study will provide valuable insights into the prevalence of MetS, ED, and accelerated aging and their co-occurrence in survivors of HSCT in childhood for a hematological malignancy. Furthermore, it will also identify previously unexplored as well as potentially modifiable risk factors for these late sequelae. The gained knowledge will inform the optimization of surveillance guidelines for long-term follow-up care of these survivors. In addition, the findings will help to develop health behavioral and potentially anti-inflammatory interventions aimed at mitigating these severe late effects in survivors at risk, ultimately improving their quality of life. Through this knowledge, the MetVasA study aims to significantly improve the well-being of these people.

## Data Availability

The datasets generated during this study will be available from the corresponding author on reasonable request. The data are not publicly available due to privacy and ethical restrictions.

## Abbreviations

6MWT: 6-minute-walking test
CVD: cardiovascular disease
DCCSS: Dutch Childhood Cancer Survivor Study
DXA: dual-energy x-ray absorptiometry
ED: endothelial dysfunction
GvHD: graft-versus-host disease
HSCT: hematopoietic stem cell transplantation
MetS: metabolic syndrome
MetVasA: Metabolic Syndrome, Vascular Damage, and Accelerated Aging
RHI: reactive hyperemia index
SRT: steep ramp test
WC: waist circumference
